# Relationships with caregivers and mental health outcomes among adolescents living with HIV: a prospective cohort study in South Africa

**DOI:** 10.1186/s12889-020-10147-z

**Published:** 2021-01-20

**Authors:** Yulia Shenderovich, Mark Boyes, Michelle Degli Esposti, Marisa Casale, Elona Toska, Kathryn J. Roberts, Lucie Cluver

**Affiliations:** 1 Centre for Evidence-Based Intervention, Department of Social Policy and Intervention, University of Oxford, Oxford, UK; 2 School of Psychology, Faculty of Health Sciences, Curtin University, Perth, Australia; 3 School of Public Health, University of the Western Cape, Cape Town, South Africa; 4 Centre for Social Science Research, University of Cape Town, Cape Town, South Africa; 5 Department of Sociology, University of Cape Town, Cape Town, South Africa; 6 Institute for Global Health, University College London, London, UK; 7 Department of Psychiatry and Mental Health, University of Cape Town, Cape Town, South Africa

**Keywords:** Mental health, Depression, Anxiety, Adolescents, HIV, Parenting

## Abstract

**Background:**

Mental health problems may impact adherence to anti-retroviral treatment, retention in care, and consequently the survival of adolescents living with HIV. The adolescent-caregiver relationship is an important potential source of resilience. However, there is a lack of longitudinal research in sub-Saharan Africa on which aspects of adolescent-caregiver relationships can promote mental health among adolescents living with HIV. We draw on a prospective longitudinal cohort study undertaken in South Africa to address this question.

**Methods:**

The study traced adolescents aged 10–19 initiated on antiretroviral treatment in government health facilities (*n* = 53) within a health district of the Eastern Cape province. The adolescents completed standardised questionnaires during three data collection waves between 2014 and 2018. We used within-between multilevel regressions to examine the links between three aspects of adolescent-caregiver relationships (caregiver supervision, positive caregiving, and adolescent-caregiver communication) and adolescent mental health (depression symptoms and anxiety symptoms), controlling for potential confounders (age, sex, rural/urban residence, mode of infection, household resources), *n*=926 adolescents.

**Results:**

Improvements in caregiver supervision were associated with reductions in anxiety (0.98, 95% CI 0.97–0.99, *p*=0.0002) but not depression symptoms (0.99, 95% CI 0.98–1.00, *p*=.151), while changes in positive caregiving were not associated with changes in mental health symptoms reported by adolescents. Improvements in adolescent-caregiver communication over time were associated with reductions in both depression (IRR=0.94, 95% CI 0.92–0.97, *p*<.0001) and anxiety (0.91, 95% CI 0.89–0.94, *p*<.0001) symptoms reported by adolescents.

**Conclusions:**

Findings highlight open and supportive adolescent-caregiver communication and good caregiver supervision as potential factors for guarding against mental health problems among adolescents living with HIV in South Africa. Several evidence-informed parenting programmes aim to improve adolescent-caregiver communication and caregiver supervision, and their effect on depression and anxiety among adolescents living with HIV should be rigorously tested in sub-Saharan Africa. How to improve communication in other settings, such as schools and clinics, and provide communication support for caregivers, adolescents, and service providers through these existing services should also be considered.

**Supplementary Information:**

The online version contains supplementary material available at 10.1186/s12889-020-10147-z.

## Background

Adolescence and early adulthood are a time of learning and growth. This is also the life stage when most mental health disorders emerge [1, 2], alongside vast physical, emotional, and social changes [3]. While peer relationships become increasingly important in adolescence, caregivers remain a key potential source of support for adolescent health and wellbeing [4]. Adolescent-caregiver relationships are an important dimension of family functioning [5] and can affect adolescent mental health [6, 7].

Adolescents living with HIV (ALHIV) often are at a heightened risk of mental health challenges compared to their peers [8–10]. Poor mental health may reduce adolescent adherence to anti-retroviral treatment, retention in care, and increase risk behaviours, negatively affecting life trajectories and survival [11–14]. It is estimated that there are 1.6 million ALHIV aged 10–19 years globally, most of whom live in sub-Saharan Africa [15].

There is, however, limited evidence that can inform effective mental health provision for ALHIV in the region. A set of global systematic reviews supported by the World Health Organization and UNICEF *Helping Adolescents Thrive* collaboration identified only three randomised controlled trials with ALHIV evaluating interventions to promote mental health, two of which were conducted in sub-Saharan Africa [16, 17], including only one intervention focused on adolescent-caregiver relationships [18].

Furthermore, while experimental studies provide insights into the overall effects of interventions [19], programme evaluations often do not examine how specific components or experiences may be linked to adolescent mental health. Observational studies can help identify the aspects of adolescent-caregiver relationships that are particularly important for ALHIV mental health to make sure these aspects are targeted in intervention design and research [20, 21]. However, a recent literature review [8] identified a lack of longitudinal observational studies on factors related to depression among ALHIV in Southern Africa. Furthermore, the review found that existing cross-sectional studies in Southern Africa have primarily recruited participants from specialist HIV clinics, limiting the generalizability of the findings [8].

In the current study, we examine whether three aspects of adolescent-caregiver relationships are related to depression and anxiety symptoms among ALHIV in the Eastern Cape Province of South Africa. Anxiety and depression often co-occur but are distinct, and co-morbid anxiety can worsen depression [22], thus considering approaches that may be able to address both is important. We draw on the region’s first large-scale ALHIV cohort study, conducted in 2014–2018, that identified all ALHIV initiated on care within a health district and traced them into the community, to avoid a sample biased towards those engaged in care [23]. Adolescence is a highly dynamic time [3], and during the study period some young people in the cohort experienced changes to their family environments such as moving households and changing caregivers [24]. In this study we, therefore, focus on examining changes in the adolescent-caregiver relationships and corresponding changes in depression and anxiety symptoms, utilising the cohort’s three-wave repeated measures.

## Methods

### Location and participants

The study took place in the South African Eastern Cape province, a historically disadvantaged region with poor infrastructure [25–27]. We identified all 53 public community health centres, primary clinics and hospitals providing HIV treatment to adolescents in the Buffalo City Health District in the Eastern Cape. The study area was selected in consultation with government partners, UN agencies, and non-governmental organisations. In each healthcare facility, all files (paper and electronic) were reviewed to identify young people who had initiated HIV treatment and were between 10 and 19 years of age. These adolescents were traced to 180 communities. The study team recruited 1046 adolescents living with HIV into the study, who were interviewed at baseline in 2014–15. These adolescents represented 90% of all 1176 patient files found. There were no statistically significant differences between adolescents who were and were not recruited on age, sex, and rural/urban residence [28].

**Fig. 1:**
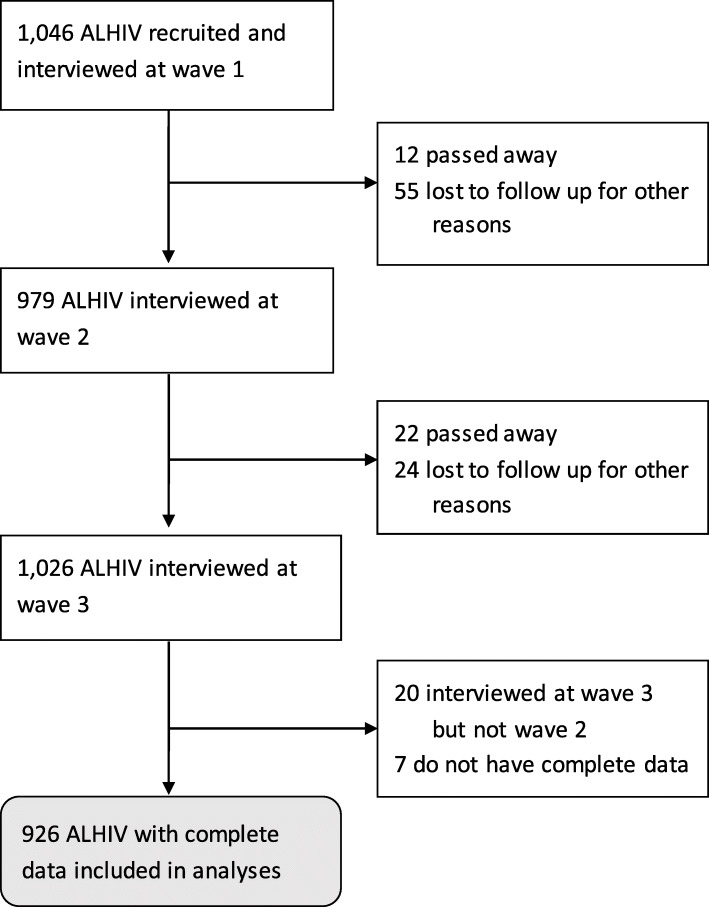
Flow diagram of study sample

All adolescents who had given consent to be re-approached were invited to be interviewed again in 2016–17 and 2017–18. Out of 1046 ALHIV at baseline, 93% were followed up at wave 2 of the study, and 98% at wave 3. Among reasons for attrition, some participants could not be traced, or were not willing or available to participate, and 34 (3%) young people died during the study period. In addition, seven participants were not included in the current analyses due to incomplete data. Our analyses focus on the 926 participants interviewed across all three waves, using a complete case analysis (see Fig. 1 for the flow diagram).

Ethical approvals were given by the University of Cape Town (CSSR 2013/4), Oxford University (CUREC2/12–21), Provincial Departments of Health and Education and all participating healthcare facilities. All young people and their primary caregivers provided written informed consent, also read aloud in cases of low literacy. The study did not provide financial incentives. Based on the recommendation of the study’s adolescent advisory group, the young people were provided with a snack and small gift pack including toiletries and school stationery, regardless of interview completion.

Participants who were experiencing serious harm or were at risk of harm (e.g., from sexual violence, suicidal attempts, or symptomatic untreated tuberculosis) received support from the study team to engage with relevant healthcare and psychosocial services. To reduce the risk of HIV-related stigma, the study was presented in the community as a study on adolescent health. Furthermore, the study included a large group of community peers. Some ALHIV were not yet aware of their HIV status at the time of the interview (32% of the current sample at wave one, reducing to 12% at wave three, as the cohort aged). To avoid unintentional disclosure, the research team implemented a coding system for research staff and a corresponding pattern in the questionnaire. For example, the adolescents who did not know their status were asked questions about their “illness” rather than about “HIV”. The research team also took a case management approach to all participants whose disclosure status did not match, for example, if an adolescent reported knowing their HIV status but their caregiver did not know, the adolescents were offered psychosocial support. Even in cases when caregivers noted that the adolescent did know their status, the research team confirmed this knowledge through three self-reported questions (considering the young age of participants): knowledge of HIV status, knowledge that they were taking ART, and knowledge that ART were for HIV-positive status. In cases of disclosure discrepancy, the research team supported participants and their caregivers to access psychosocial support at local NGOs or referrals to highly recommended counsellors in regional hospitals and better-resourced larger health facilities.

### Measures

Questionnaires were translated into Xhosa and translations were checked by back-translation. Pre-piloting was conducted locally with 25 adolescents living with HIV. All study questionnaires are available online: http://www.youngcarers.org.za/youthpulse. See Supplementary Materials for the items and response options of all the study measures used in this analysis.

Adolescents chose their language of participation (Xhosa or English). Questionnaires were administered by trained research assistants using electronic tablets, in a location selected by the participants (e.g., home, school, outdoors). Completing the questionnaire lasted 60–90 min, and research assistants encouraged participants to take breaks where needed to help participant concentration.

#### Outcome variables

*Depression symptoms* (past 2 weeks) were measured using the Child Depression Inventory short form (CDI-S) 10-item version [29], Cronbach’s *α =* 0.53–0.58. CDI-S is a widely-used measure and has been used and validated in other South African populations [30–32]. CDI-S provides three response options. As the most severe responses were endorsed by a very small number of participants [33], the responses “1” and “2” were combined, generating a binary indicator that captured having no experience of the symptom (“0”) versus some experience of the symptom (“1”) in the past 2 weeks. *Anxiety symptoms* (past month) were measured with the widely-used Children’s Manifest Anxiety Scale – Revised (RCMAS) [34, 35], validated in South Africa with AIDS-affected children [36]. This study used a 14-item abbreviated version based on previous research [37], *α* = 0.79–0.83. The responses to the RCMAS items are “no” and “yes” to the experience of each symptom, coded as “0” and “1”. In both depression and anxiety measures, all symptoms had equal weight.

#### Explanatory and control variables

*Caregiver supervision* (past 2 months, α = 0.89–0.93) and *positive caregiving* (past 2 months, α = 0.89–0.93) were measured using the relevant subscales of the Alabama Parenting Questionnaire [38], an instrument widely used internationally, as well as previously in South Africa with a similar population [39]. Supervision items focus on caregiver awareness of the adolescent’s whereabouts and activities, while positive caregiving captures behaviours such as the caregiver praising the adolescent. Supervision was reverse-coded, so that a higher score indicated better supervision. *Caregiver communication* (past 2 months) was measured using an adapted version of the Child-Parent Communication Apprehension Scale for use with Young adults [40]. The scale asks about adolescent-caregiver overall communication as well as communication on sensitive issues, such as medication and sex, *α =* 0.56–0.75.

Sociodemographic factors included adolescent age, sex, urban/rural location, and the mode of HIV infection (perinatal and recently infected, see [41] on terminology). Mode of infection was determined by researchers based on the age of ARV initiation before or after 10 years of age, and further checked and re-coded based on other factors, such as parental orphanhood, HIV-infected parents, adolescent sexual history, and other factors, as reported elsewhere [42]. Household resources were measured based on access to the eight highest socially-perceived necessities for children, such as enough food and money for school fees, identified in the nationally representative South African Social Attitudes Survey [43].

### Statistical analyses

Analyses consisted of four main steps. First, we checked for baseline differences between the adolescents with complete information from three study waves and those with missing data; this was done using t-tests for continuous variables and Pearson’s chi-square tests for binary variables. Second, we examined the frequencies of all measures at each wave of data collection for the study sample. Third, we examined correlations between all variables used in our analyses. Fourth, we examined the relationship between caregiving practices and adolescent mental health (anxiety and depression) through regression analyses. We examined all family relationships as time-variant predictors [44]. Sociodemographic factors that could be potential confounders were identified through the creation of a causal framework ( [45], see Supplementary materials) and potential confounders were used as control variables in the multivariable regression model.

We used a “within-between” regression model [46–49], also known as a hybrid model, which combines many advantages of fixed and random effects models. This allows us to estimate both within- and between-person differences over time. Within-person estimates examine whether changes in adolescent-caregiver relationship correspond to changes in depression and anxiety symptoms over the three time-points within the same individual, thus controlling for all time-invariant confounders [50]. Between-person estimates examine whether differences between young people’s relationships with their caregivers explain some of the differences in depression and anxiety when measures are averaged across all three time-points.

For each characteristic of the adolescent-caregiver relationship, we use a person’s average value and time-specific deviation from this average (see equation [1]). Since our outcome is the number of symptoms, we use a count model, log-linked negative binomial, to account for overdispersion [51]. Thus, the analyses use the following model:

1 $$ \mathit{\log}\left(E\left({y}_{ti}\right)\right)={\beta}_0+{\beta}_1\left({\overline{x}}_i\right)+{\beta}_2\left({x}_{ti}-{\overline{x}}_i\right)+{\beta}_3\left({x}_{ti}\right)+{v}_{i0} $$

where E (*y*_*ti*_) represents the expected number of mental health symptoms, *β*_0_ is the intercept, ${\overline{x}}_i$ is the average of the predictor for person *i* across three timepoints, *x*_*ti*_ is a time-varying predictor for person *i* at time *t*,. Thus, *β*_1_ represents the average between-person effect and *β*_2_ represents the average within-person effect, and *v*_*i*0_ is a random person-level intercept, assumed to be normally distributed. Since this is an age-heterogenous cohort and given participant mobility, we also included time-varying measures of age, household necessities and rural/urban residence as control variables (*β*_3_). For time-invariant control variables (gender and mode of HIV infection), a single value is included from baseline. Variance is defined as follows:

2 $$ \mathit{\operatorname{var}}\left({y}_{ti}\right)=E\left({y}_{ti}\right)+\alpha {\left(E\left({y}_{ti}\right)\right)}^2 $$

where *α* is a dispersion parameter.

All parameters are estimated by maximum likelihood and with robust standard errors, clustered at the level of the individual. Regression outputs are presented as incidence rate ratios (IRRs) [52]. As reference, IRR=1.00 indicates no difference in the outcome based on the values of the explanatory variable, IRR< 1.00 indicates a decreased rate of the outcome, and IRR> 1.00 – an increased rate. Stata 14.2 was used for all analyses, the code can be found on the project page (https://osf.io/fwy2d/).

## Results

### Study sample and attrition

Comparing the 926 young people retained in the study and those who were not, we did not find differences on most baseline measures (see Supplementary Materials Table S1 for details). However, we observed that those who were not retained across all time points were approximately a year older at baseline (retained participant age mean=13.55 (SD=2.88), lost-to-follow-up m=14.57 (2.87), *p*< 0.001), reported higher baseline depression symptoms (retained m=0.84 (SD=1.30), lost-to-follow-up m=1.16 (SD=1.56), *p*=0.020), lower level of caregiver supervision (retained m=33.89 (SD=8.92), lost-to-follow-up m=32.08 (SD=9.61, *p*=0.038) and of caregiver communication (retained m=13.80 (SD=2.79), lost-to-follow-up m=13.13 (SD=3.52, *p=0.017*).

### Descriptive statistics

The characteristics of the analytical sample at each study wave are presented in Table 1 below. Study participants were on average 13.6 years old at baseline (55% female). For the majority, their primary caregiver was not a biological parent (usually grandmother or aunt), therefore we refer to primary caregiver as any adult living in the same household that takes care of an adolescent. The overall prevalence of reported symptoms of depression and anxiety, on average, decreased across the three study waves in our sample.

**Table 1:** Sample description (*N*=926)

Variable	Wave 1	Wave 2	Wave 3	Range of scales
Gender (female), N (%)	509 (55)			
Age (years), mean (SD)	13.55 (2.88)	15.06 (2.88)	16.25 (2.90)	N/A
Rural, N (%)	246 (27)	228 (25)	221 (24)	N/A
Mode of infection (recently infected), N (%)	197 (21)			N/A
Primary caregiver not biological parent, N (%)	519 (56)	548 (59)	552 (60)	N/A
Orphan, N (%)	544 (59)	558 (60)	637 (69)	N/A
Necessities household can afford out of 8, mean (SD)	6.37 (1.81)	5.55 (2.26)	5.69 (2.29)	0–8
Depression symptoms, mean (SD)	0.84 (1.30)	0.60 (1.11)	0.48 (1.01)	0–10
Anxiety symptoms, mean (SD)	2.15 (2.62)	0.87 (1.89)	0.67 (1.55)	0–14
Caregiver supervision scale, mean (SD)	33.89 (8.92)	34.86 (6.58)	33.97 (7.34)	0–24
Positive caregiving scale, mean (SD)	19.82 (4.88)	18.95 (5.23)	18.41 (5.30)	0–40
Caregiver communication scale, mean (SD)	13.80 (2.79)	13.81 (2.64)	14.01 (2.95)	0–20

Orphan defined as someone with one or both biological parents who have passed away. Depression items coded as yes/no for each of the 10 symptoms.

The correlation of anxiety and depression symptoms was 0.43 (see Supplementary Materials Table S2 for the correlation matrix), so it was informative to analyse them as separate outcomes. VIF values for the variables included in the models ranged between 1.03 and 1.48, suggesting multicollinearity was not a concern.

### Regression analyses

First, we ran single-predictor regressions (see Supplementary Materials Table S3), followed by multivariable regressions to examine within- and between-person differences in mental health, explained by adolescent-caregiver relationships (see Table 2 below), while controlling for potential confounders. We found that changes in caregiver supervision were related to lower anxiety (0.98, 95% CI 0.97–0.99, *p*=0.0002) but not depression symptoms (0.99, 95% CI 0.98–1.00, *p*=.151). We also observed that young people with higher levels of caregiver supervision reported lower depression (0.97, 95% CI 0.96–0.99, *p*=.0002) and anxiety symptoms (0.97, 95% CI 0.95–0.99, *p*=.0001). Changes in positive caregiving were not associated with changes in depression and anxiety, while the young people who reported, on average, greater levels of positive caregiving also reported fewer symptoms of depression (IRR=0.95, 95% CI 0.93–0.97, *p*<.0001) and anxiety (0.96; 95% CI 0.94–0.98, *p*=.0003). Improvements in communication with caregivers corresponded to reductions in depression (0.94, 95% CI 0.92–0.97, *p*<.0001) and anxiety (0.91, 95% CI 0.89–0.94, *p*<.0001) symptoms over time. Similarly, young people who experienced better communication with caregivers on average across all three timepoints reported fewer depressive symptoms (IRR=0.92, 95% CI 0.89–0.96, *p*=.0002) and anxiety symptoms (0.89, 95% CI 0.85–0.93, *p*<.0001).

**Table 2:** Multivariable regressions (*n*=926, 3 data waves)

Explanatory variables	Depression symptoms				Anxiety symptoms			
	IRR	95% CI		*p*-val.	IRR	95% CI		*p*-val.
Caregiver supervision – within	0.99	0.98	1.00	0.151	0.98	0.97	0.99	0.0002
Caregiver supervision – between	0.97	0.96	0.99	0.0002	0.97	0.95	0.99	0.0001
Positive caregiving – within	1.00	0.98	1.01	0.559	1.00	0.98	1.01	0.626
Positive caregiving – between	0.95	0.93	0.97	< 0.0001	0.96	0.94	0.98	0.0003
Caregiver communication – within	0.94	0.92	0.97	< 0.0001	0.91	0.89	0.94	< 0.0001
Caregiver communication - between	0.92	0.89	0.96	0.0002	0.89	0.85	0.93	< 0.0001

Control variables in model: age, gender, mode of infection, rural, timepoint, household necessities. The coefficients for control variables are presented in the Supplementary Materials Table S4. It was not possible to test random slopes due to lack of model conversion with random slopes.

### Sensitivity analysis

We have also tested our results with two additional control variables, adolescent orphanhood and living with a caregiver who is not a biological parent, which did not affect our findings (see Supplementary Materials Table S5 for results including these additional controls).

## Discussion

We have examined how adolescent relationships with caregivers are related to anxiety and depression symptoms within a cohort of adolescents living with HIV. We found that a greater level of open and supportive communication with caregivers was related to decreases in both adolescent depression and anxiety symptoms among adolescents living with HIV. Supportive and open communication, therefore, may be an important source of support as adolescents navigate biological, emotional, and social changes, in the context of living with HIV. Furthermore, improvements in caregiver supervision, which may indicate a closer adolescent-caregiver relationship, were associated with reductions in adolescent anxiety symptoms.

One strategy to support adolescent-caregiver relationships are parenting and family strengthening programmes [53–55]. Several programmes have been developed or tailored for families with ALHIV [56] and adolescents affected by HIV [57, 58]. For example, the Vuka programme [18] is a group-based intervention for adolescent-caregiver pairs that addresses adolescent-caregiver communication, strategies for adolescent safety in the community, and HIV knowledge and disclosure, among other topics. While a small-scale pilot randomised evaluation of Vuka in South Africa did not find statistically significant intervention effects of the programme on adolescent mental health, the pooled data from South Africa, US, and Argentina pilots suggested a trend of improvement in the emotional wellbeing of participating ALHIV, compared to the control groups [56, 59, 60]. Two small pilot trials of the adapted Family Strengthening Intervention programme with HIV-affected families in Rwanda found intervention effects on reducing adolescent depression symptoms [57, 61]. Other parenting interventions have targeted adolescent-caregiver communication as a means of reducing the risk of new adolescent HIV infections [62, 63], including within PEPFAR-USAID supported programmes delivered in Sub-Saharan Africa focusing on adolescent girls and young women. Adolescent-caregiver communication has also been identified as a key aspect of many violence prevention parenting programmes [64].

Universally offered parenting programmes may support mental health among adolescents by strengthening adolescent-caregiver relationships and communication, without specifically focusing on families of adolescents living with or affected by HIV and thus reduce the risk of stigma or accidental disclosure [65], which may be one of the multiple decision-making considerations in choosing a programme [66]. A study in South Africa, nested within a randomised trial, found that both ALHIV and HIV-positive caregivers were as likely to attend a parenting programme as other community members [67], and families where caregivers lived with HIV had similar outcomes to those who did not [68]. However, targeted programmes for families affected by HIV can provide families with HIV-related psychoeducation on topics such as communicating about HIV, as well as strategies for antiretroviral medication adherence and other health behaviours. Based on our findings, adolescent-caregiver communication and caregiver monitoring might be important aspects of existing parenting programmes in relation to adolescent mental health as an outcome. In 2020, the importance of good communication with young people has been made even more salient in the context of dealing with a new health challenge of the COVID-19 pandemic.

Looking at the composition of our community-traced sample, around 80% of adolescents were identified as infected perinatally. Different modes of HIV infection can pose different psychological challenges – for instance, recently infected adolescents often encounter worse stigma in healthcare settings. On the other hand, adolescents infected at birth have or had a caregiver living with HIV, which may create additional stress and stigma, but also provide a shared experience and knowledge of living with HIV and navigating the healthcare system [42, 69]. Due to caregiver HIV and other reasons, over half of our participants have lost one or both biological parents. Around 45–50% of adolescents in our sample (depending on study round) were both orphaned and did not have a biological parent as their primary caregiver – many of these adolescents were cared for by grandparents or extended family. Research from the region suggests that intergenerational households may experience particular communication and relationship challenges [70]. In our sample, open communication reported by orphaned adolescents and those whose primary caregiver was not a biological parent were lower than among their peers. Nevertheless, while accounting for family structures, as well as mode of infection, improvements in communication were associated with a decrease in both depression and anxiety symptoms.

ALHIV must navigate relationships not only with their caregivers, but also with other adults, such as their healthcare providers and teachers. Cross-sectional analyses from the baseline of the current cohort suggest that kind communication from healthcare providers is associated with reduced sexual risk-taking and improved retention in HIV care [28, 71]. Recent guidance for healthcare workers provides examples of good communication practices for talking to adolescents about illness [72]. Clinics and schools may serve as channels to promote supportive communication between ALHIV and service providers in these settings, as well as to support adolescent-caregiver communication by offering relevant advice to families [73, 74]. Additional research is needed to test the effect of improved adolescent-provider communication on adolescent mental health, as well as whether providers can support adolescent-caregiver communication.

Adolescents can also be supported to strengthen their own communication skills. In a systematic review of universally-delivered psychosocial interventions for adolescents, programme components targeting interpersonal skills – defined as the ability to develop strong and close relationships with others – were consistently linked to multiple positive adolescent outcomes [75]. In addition to the direct guidance and training for adolescents, caregivers, and service providers, a potential area for further research is modelling examples of good communication with adolescents in public forums, such as radio and television programming [76, 77].

In our regression analyses, we found stronger links of adolescent-caregiver relationships and adolescent mental health between individuals than within individuals over time. Since the within-person analyses account for unmeasured stable characteristics such as personality and are not biased by such time-invariant confounders, these unmeasured variables may be driving some of the between-person differences [50]. In addition, within-person analyses tend to be less well-powered since they draw on variation in explanatory and outcome variables [50]. Another possibility may be that consistent exposure to supportive and engaged caregivers is important for better mental health, whereas within-person differences focus on change. Research drawing on this and another cohort in South Africa found the importance of consistent supportive circumstances and services for healthy adolescent development [78].

There are both strengths and limitations of the current analyses. These analyses used repeated measurements, which allowed us to separate and examine the within- and between-person variation. While we have utilised within-person variation to account for potential time-invariant confounding [19], our analyses do not provide causal certainty – for instance, we cannot account for time-variant unmeasured confounders. All the study measures have been locally piloted and previously used in South Africa with similar age groups. Nevertheless, using self-report measures of adolescent-caregiver relationships and mental health introduces the risk of method overlap bias, as adolescents who are more anxious and depressed may perceive their relationships more negatively. Furthermore, some of the scales (depression and communication) had poor internal reliability, which may be in part due to their brevity [79]. We focus on one of many aspects of adolescents’ life that can affect mental health. The analyses are based on a study sampling all adolescents initiated on ART treatment in an entire health district and had high retention rates. However, we were not able to recruit and trace all adolescents, so the analyses may have excluded some of the most vulnerable participants and therefore may not generalise to everyone. While in our study most adolescents appeared to be perinatally infected, recently infected adolescents are also a key group affected by HIV in the region [68]. Additional research could examine if there are any differences in the types of family and psychosocial support that may benefit adolescents infected at birth and recently infected adolescents.

## Conclusions

Our analyses, drawing on longitudinal data from a community-traced cohort, highlight the potential of open and supportive adolescent-caregiver communication for reducing both depression and anxiety symptoms among adolescents living with HIV. Promising areas for intervention and research on strengthening adolescent-caregiver communication include family-based parenting programmes and communication support through other channels, such as schools and clinics.

## Supplementary Information

**Additional file 1.** Supplementary tables and list of questionnaire items.

## Data Availability

Data generated and analysed during the current study will be made available following full anonymisation in accordance with the study’s data management processes, but are available from the corresponding author for non-commercial use based on data sharing policies and processes outlined here: http://www.mzantsiwakho.org.za/wp-content/uploads/2016/05/MW_DataSharingAccessPolicy_final.pdf

## Abbreviations

ALHIV: Adolescents living with HIV
ART: Anti-retroviral treatment
HIV: Human immunodeficiency virus
