# Supplementary material for: Relationships with caregivers and mental health outcomes among adolescents living with HIV: a prospective cohort study in South Africa
Source: BMC Public Health. 2021 Jan 20;21:172. doi: 10.1186/s12889-020-10147-z (PMC7816135; doi:10.1186/s12889-020-10147-z)
Supplement: Supplementary file 1 — Additional file 1. Supplementary tables and list of questionnaire items. [file 12889_2020_10147_MOESM1_ESM.docx]

**Supplementary materials**

Figure S1. Conceptual framework


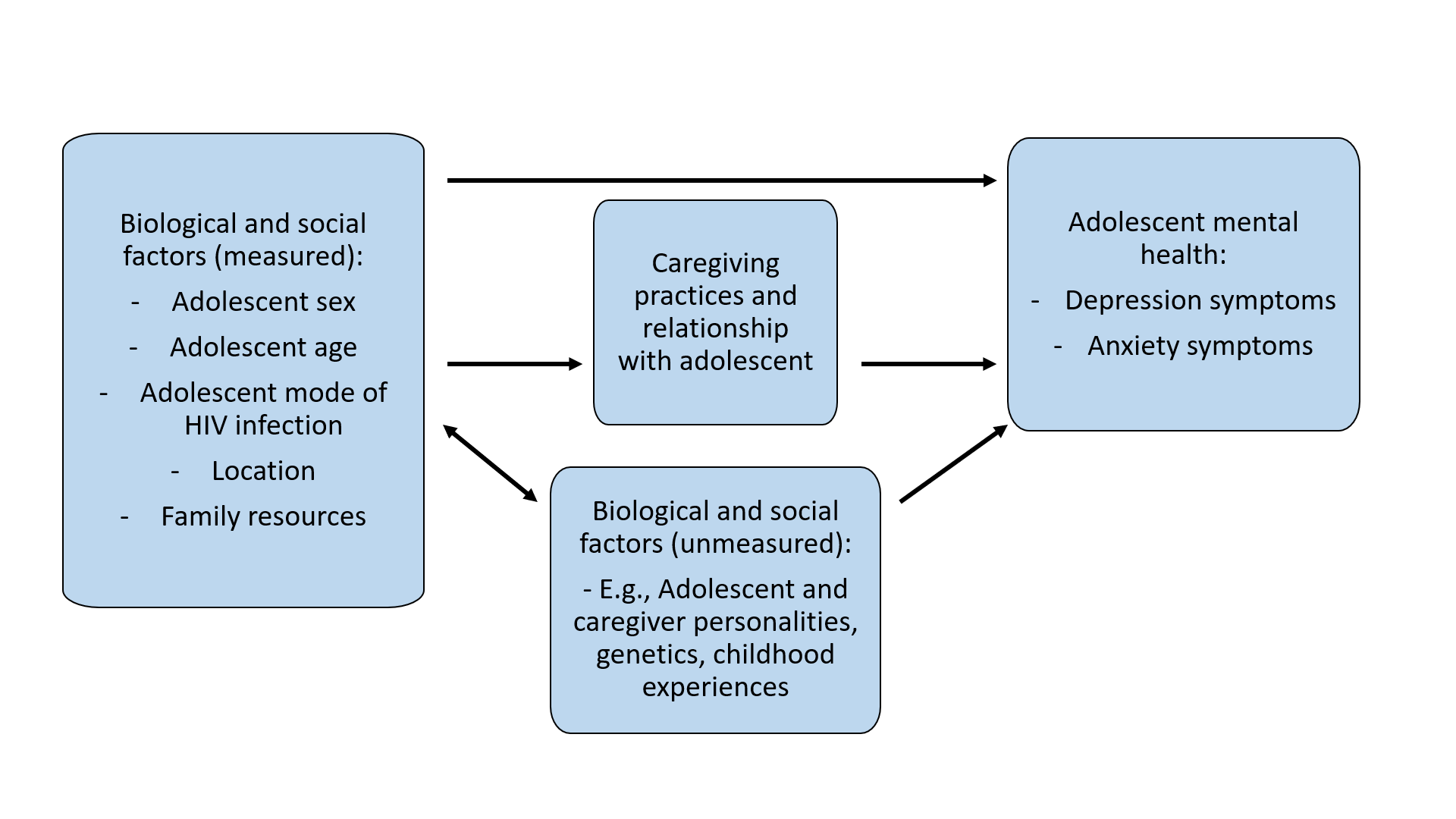


Table S1. Baseline characteristics of study participants by loss to follow up.

| Baseline characteristic (wave 1) | Included  (n=926) | Lost to follow-up/ not included (n=120) | p-value for difference between groups |
| --- | --- | --- | --- |
| Gender (female), N (%) | 509 (55) | 67 (56) | 0.860 |
| Age, mean (SD) | 13.55 (2.88) | 14.57 (2.87) | <0.001 |
| Rural, N (%) | 246 (27) | 25 (21) | 0.170 |
| Mode of infection (post-natal) , N (%) | 197 (21) | 25 (23) | 0.690 |
| Orphan, N (%) | 544 (59) | 72 (60) | 0.790 |
| Primary caregiver not biological parent, N (%) | 519 (56) | 59 (49) | 0.150 |
| Household necessities, mean (SD) | 6.37 (1.81) | 6.47 (1.77) | 0.560 |
| Depression symptoms, mean (SD) | 0.84 (1.30) | 1.16 (1.56) | 0.014 |
| Anxiety symptoms, mean (SD) | 2.15 (2.62) | 2.33 (2.87) | 0.490 |
| Caregiver supervision, mean (SD) | 33.89 (8.92) | 32.08 (9.61) | 0.038 |
| Positive caregiving, mean (SD) | 19.82 (4.88) | 19.52 (4.82) | 0.540 |
| Caregiver communication, mean (SD) | 13.80 (2.79) | 13.13 (3.52) | 0.017 |

Table S2. Bivariate correlations for all the measures included in the regression model (n=926)

|  | Depression symptoms | Anxiety symptoms | Caregiver supervision | Positive caregiving | Caregiver communication | Household necessities | Gender | Age | Mode of infection |
| --- | --- | --- | --- | --- | --- | --- | --- | --- | --- |
| Anxiety symptoms | 0.43 |  |  |  |  |  |  |  |  |
| Caregiver supervision | -0.14 | -0.17 |  |  |  |  |  |  |  |
| Positive caregiving | -0.13 | -0.11 | 0.14 |  |  |  |  |  |  |
| Caregiver communication | -0.18 | -0.20 | 0.07 | 0.20 |  |  |  |  |  |
| Household necessities | -0.07 | -0.05 | 0.12 | 0.26 | 0.12 |  |  |  |  |
| Gender (female) | 0.05 | 0.07 | 0.05 | 0.02 | 0.01 | -0.02 |  |  |  |
| Age | 0.10 | 0.02 | -0.31 | -0.19 | -0.03 | -0.19 | 0.14 |  |  |
| Mode of infection (post-natal) | 0.11 | 0.11 | -0.26 | -0.11 | -0.06 | -0.10 | 0.17 | 0.50 |  |
| Rural | 0.00 | 0.02 | 0.03 | -0.14 | -0.03 | -0.03 | 0.03 | 0.01 | 0.03 |

Table S3. Single-predictor regressions (n=926)

|  | Depression (number of symptoms) | | | | Anxiety (number of symptoms) | | | |
| --- | --- | --- | --- | --- | --- | --- | --- | --- |
| *Explanatory variables* | IRR | 95% CI | | p-value | IRR | 95% CI | | p-value |
| Caregiver supervision - *within* | 0.99 | 0.98 | 1.00 | 0.047 | 0.97 | 0.96 | 0.98 | <0.001 |
| *Between* | 0.95 | 0.94 | 0.97 | <0.0001 | 0.96 | 0.94 | 0.97 | <0.001 |
| Positive caregiving - *within* | 0.99 | 0.98 | 1.01 | 0.222 | 1.00 | 0.98 | 1.01 | 0.492 |
| *Between* | 0.92 | 0.90 | 0.94 | <0.0001 | 0.93 | 0.91 | 0.95 | <0.001 |
| Caregiver communication - within | 0.94 | 0.91 | 0.96 | <0.0001 | 0.93 | 0.90 | 0.96 | <0.001 |
| *Between* | 0.88 | 0.85 | 0.92 | <0.0001 | 0.88 | 0.85 | 0.91 | <0.001 |
| Household necessities can afford | 0.96 | 0.93 | 0.99 | 0.013 | 0.96 | 0.93 | 1.00 | 0.041 |
| Age | 1.04 | 1.01 | 1.06 | 0.003 | 1.01 | 0.99 | 1.03 | 0.435 |
| Gender (female) | 1.16 | 1.00 | 1.34 | 0.053 | 1.29 | 1.12 | 1.48 | 0.004 |
| Mode of infection (post-natal) | 1.44 | 1.20 | 1.73 | 0.0001 | 1.50 | 1.28 | 1.76 | <0.001 |
| Rural | 1.01 | 0.85 | 1.19 | 0.934 | 1.04 | 0.82 | 1.31 | 0.762 |
| Wave 1 | Ref |  |  |  | Ref |  |  |  |
| Wave 2 | 0.69 | 0.61 | 0.80 | <0.0001 | 0.35 | 0.30 | 0.41 | <0.001 |
| Wave 3 | 0.56 | 0.48 | 0.64 | <0.0001 | 0.27 | 0.23 | 0.32 | <0.001 |

*Note: For the adolescent-caregiver relationship factors, both within- and between-effects are included in the same model. Rural-anxiety is modelled using Poisson link since negative binomial did not converge. All models use robust standard errors.*

Table S4. Multivariable regression (all parameters for the Table 2 in text), n=926

|  | Depression (number of symptoms) | | | | Anxiety (number of symptoms) | | | |
| --- | --- | --- | --- | --- | --- | --- | --- | --- |
| *Explanatory variables* | IRR | 95% CI | | p-value | IRR | 95% CI | | p-value |
| Caregiver supervision - *within* | 0.99 | 0.98 | 1.00 | 0.151 | 0.98 | 0.97 | 0.99 | <0.001 |
| *Between* | 0.97 | 0.96 | 0.99 | <0.001 | 0.97 | 0.95 | 0.99 | <0.001 |
| Positive caregiving - *within* | 1.00 | 0.98 | 1.01 | 0.559 | 1.00 | 0.98 | 1.01 | 0.626 |
| *Between* | 0.95 | 0.93 | 0.97 | <0.001 | 0.96 | 0.94 | 0.98 | <0.001 |
| Caregiver communication - within | 0.94 | 0.92 | 0.97 | <0.001 | 0.91 | 0.89 | 0.94 | <0.001 |
| *Between* | 0.92 | 0.89 | 0.96 | <0.001 | 0.89 | 0.85 | 0.93 | <0.001 |
| Household necessities | 0.98 | 0.95 | 1.01 | 0.137 | 0.98 | 0.94 | 1.01 | 0.193 |
| Age (years) | 1.04 | 1.01 | 1.07 | 0.007 | 1.02 | 0.99 | 1.05 | 0.197 |
| Gender (female) | 1.17 | 1.01 | 1.35 | 0.040 | 1.33 | 1.14 | 1.55 | <0.001 |
| Mode of infection (post-natal) | 0.89 | 0.73 | 1.09 | 0.259 | 0.98 | 0.79 | 1.21 | 0.843 |
| Rural | 0.89 | 0.76 | 1.04 | 0.154 | 0.94 | 0.80 | 1.11 | 0.478 |
| Wave 1 | Ref |  |  |  | Ref |  |  |  |
| Wave 2 | 0.64 | 0.55 | 0.74 | <0.001 | 0.33 | 0.28 | 0.39 | <0.001 |
| Wave 3 | 0.49 | 0.41 | 0.59 | <0.001 | 0.24 | 0.20 | 0.30 | <0.001 |

*All models use robust standard errors.*

Table S5. Multivariable regression with additional control variables, n=926

|  | Depression (number of symptoms) | | | | Anxiety (number of symptoms) | | | |
| --- | --- | --- | --- | --- | --- | --- | --- | --- |
| *Explanatory variables* | IRR | 95% CI | | p-value | IRR | 95% CI | | p-value |
| Caregiver supervision - *within* | 0.99 | 0.98 | 1.00 | 0.149 | 0.98 | 0.97 | 0.99 | <0.001 |
| *Between* | 0.97 | 0.96 | 0.99 | <0.001 | 0.97 | 0.95 | 0.98 | <0.001 |
| Positive caregiving - *within* | 1.00 | 0.98 | 1.01 | 0.555 | 1.00 | 0.98 | 1.01 | 0.618 |
| *Between* | 0.95 | 0.93 | 0.98 | <0.001 | 0.96 | 0.94 | 0.98 | <0.001 |
| Caregiver communication - within | 0.94 | 0.92 | 0.97 | <0.001 | 0.91 | 0.89 | 0.94 | <0.001 |
| *Between* | 0.92 | 0.89 | 0.96 | <0.001 | 0.89 | 0.85 | 0.93 | <0.001 |
| Household necessities | 0.98 | 0.95 | 1.01 | 0.138 | 0.98 | 0.94 | 1.01 | 0.195 |
| Age (years) | 1.04 | 1.01 | 1.07 | 0.017 | 1.02 | 0.99 | 1.05 | 0.263 |
| Gender (female) | 1.17 | 1.01 | 1.35 | 0.037 | 1.33 | 1.14 | 1.55 | <0.001 |
| Mode of infection (post-natal) | 0.91 | 0.74 | 1.12 | 0.382 | 0.99 | 0.80 | 1.23 | 0.929 |
| Primary caregiver - biological parent | 1.01 | 0.87 | 1.16 | 0.944 | 1.03 | 0.86 | 1.22 | 0.769 |
| Adolescent is an orphan | 1.09 | 0.93 | 1.27 | 0.280 | 1.06 | 0.89 | 1.27 | 0.519 |
| Rural | 0.89 | 0.76 | 1.05 | 0.155 | 0.94 | 0.80 | 1.12 | 0.497 |
| Wave 1 | Ref |  |  |  | Ref |  |  |  |
| Wave 2 | 0.64 | 0.55 | 0.74 | <0.001 | 0.33 | 0.28 | 0.39 | <0.001 |
| Wave 3 | 0.49 | 0.41 | 0.59 | <0.001 | 0.24 | 0.20 | 0.30 | <0.001 |

*All models use robust standard errors.*

**Questionnaire items and response options**

Depression items

1. Frequency of sadness: How have you felt in the last 2 weeks?

- I am sad once in a while
- I am sad many times
- I am sad all the time

1. Feelings about appearance: How have you felt in the last 2 weeks?

- I look OK
- There are some bad things about my looks
- I look ugly

1. Feelings toward self: How have you felt in the last 2 weeks?

- I like myself
- I do not like myself
- I hate myself

1. Frequency of loneliness: How have you felt in the last 2 weeks?

- I do not feel alone
- I feel alone often
- I feel alone all the time

1. Self-evaluation: How have you felt in the last 2 weeks?

- I do most things OK
- I do many things wrong
- I do everything wrong

1. Friends: How have you felt in the last 2 weeks?

- I have enough friends
- I have some friends but wish I had more
- I don’t have any friends

1. Frequency of crying: How have you felt in the last 2 weeks?

- I feel like crying once in a while
- I feel like crying many days
- I feel like crying every day

1. Feelings of love: How have you felt in the last 2 weeks

- I am sure that somebody loves me
- I am not sure if anybody loves me
- Nobody really loves me

1. Bothered frequency: How have you felt in the last 2 weeks?

- Things bother me once in a while
- Things bother me many times
- Things bother me all the time

1. Personal outlook: How have you felt in the last 2 weeks?

- Things will work out for me OK
- I am not sure if things will work out for me
- Nothing will ever work out for me

Anxiety items

1. A lot of people are against me.
2. I am nervous.
3. I feel alone even when there are people with me.
4. I feel that others do not like the way I do things.
5. I have bad dreams.
6. I often worry about something bad happening to me.
7. I wake up scared some of the time.
8. I worry a lot of the time.
9. I worry about what is going to happen.
10. I worry about what my carers will say to me.
11. I worry about what other people think about me.
12. I worry when I go to bed at night.
13. It is hard for me to get to sleep at night.
14. Other children are happier than I am.

Response options: yes/no

**Caregiver communication**

We’d like to know more about how you feel talking to your parents or caregivers. Could you tell us about how much you’ve shared with your caregiver in the past two months?"

1. I have no fear in discussing problems with my parent or caregiver.
2. I am comfortable talking about sex or medication with my parents or caregivers.
3. I am relaxed with my parent or caregiver, I can talk to them openly.
4. When I talk to my parent or caregiver, I am anxious and careful about what I say. (reverse-coded)
5. I have no fear telling my parents or caregivers exactly how I feel.

Response options: Strongly disagree, Disagree, Don’t know, Agree, Strongly agree

**Positive parenting/caregiving**

1. Your parents or caregiver says you have done something well.
2. Your parent or caregiver compliments you when you have done something well.
3. Your parent or caregiver praises you for behaving well.
4. Your parent or caregiver tells you that they like it when you help out around the house.
5. Your parent or caregiver rewards or gives something extra to you for behaving well.
6. Your parents or caregivers hug you or kiss you when you have done something well.

Response options: Never, Rarely, Sometimes, Often, Always

**Caregiver monitoring and supervision**

1. You go out without a set time to be home.
2. You stay out in the evening past the time you are supposed to be at home.
3. You fail to leave a note or let your parent or caregiver know where you are going.
4. Your parent or caregiver does not know who you are friends with.
5. You go out after dark without an adult with you.
6. Your parent or caregiver gets so busy that they forget where you are and what you are doing.
7. You stay out later than you are supposed to and your caregiver doesn’t know it.
8. Your parent or caregiver leaves the house and doesn’t tell you where they are going.
9. You come home from school more than an hour past the time your parent or caregiver expects you to be home.
10. You are at home without an adult with you.

Response options: Never, Rarely, Sometimes, Often, Always

**Material necessities**

Please tick the things which you can afford at home:

- 3 meals a day
- School fees
- Visit to the doctor when you are ill, and all the medicines you need
- School uniform
- Enough clothes to keep you warm and dry
- Toiletries to be able to wash every day
- School equipment
- More than one pair of shoes
- None of these
